# Extended-Spectrum β-Lactamases in *Escherichia coli and Klebsiella* *pneumoniae* in Gulls, Alaska, USA

**DOI:** 10.3201/eid2005.130325

**Published:** 2014-05

**Authors:** Jonas Bonnedahl, Jorge Hernandez, Johan Stedt, Jonas Waldenström, Björn Olsen, Mirva Drobni

**Affiliations:** Linnaeus University, Kalmar, Sweden (J. Bonnedahl, J. Hernandez, J. Stedt, J. Waldenström);; Kalmar County Hospital, Kalmar (J Bonnedahl, J. Hernandez);; Uppsala University, Uppsala, Sweden (J. Hernandez, B. Olsen, M. Drobni)

**Keywords:** antibiotic resistance, ESBL, CTX-M, clonal expansion, Eschericha coli, Klebsiella pneumoniae, environmental, wild birds, Larus hyperboreus, Barrow Alaska USA, bacteria

**To the Editor:** Resistance to β-lactam antibacterial drugs has spread rapidly, particularly through the CTX-M β-lactamase enzymes (CTX-M) (*1*). Although CTX-Ms are geographically widely distributed, reports of extended-spectrum β-lactamase (ESBL) dissemination are few from remote regions. In 2008, we reported phenotypic resistance traits in *Escherichia coli* isolates in 8.2% of wild birds sampled in the Arctic (*2*). We sampled approximately 260 wild birds, mainly gulls and geese, but found no ESBL-harboring isolates (J. Bonnedahl et al., unpub. data). Here we report results of our 2010 study at Barrow, Alaska, USA, a follow up to our 2005 study in which we found vancomycin-resistant enterococci (VRE) with clear traits of human origin in glaucous gulls (*3*). Our findings show a remarkable change, not in VRE dissemination, which is fairly unchanged, but in the emergence of ESBLs and general resistance of *E. coli* isolates.

We collected 150 fecal samples from a population of adult gulls residing close to a landfill site. For a description of general resistance levels (*4**,**5*), susceptibility of 1 randomly selected *E. coli* isolate per sample (137 isolated from 150 samples) was tested to a set of 10 antibacterial agents. Nearly half (48%) of the 137 *E. coli* isolates were resistant to at least 1 of the drugs tested. Resistance to 1 or 2 antimicrobial agents was found in 32% and 13% of the tested isolates, respectively, and resistance to >3 was found in 3% of isolates (Technical Appendix Table).

We analyzed samples for presence of VRE (*3*). Seven (4.7%) *E. faecium* isolates were found, all of which harbored both the *vanA* and the *esp* genes (found in isolates of the CC17 lineage) (*3*). No other VRE were found.

To investigate the presence of ESBL-producing bacteria, we conducted a selective screen as described (*6*). ESBL-producing bacteria were found (*E. coli* and *K. pneumoniae*), and ESBL genes (*bla*_CTX-M_, *bla*_SHV_, and *bla*_TEM_) in ESBL-positive isolates were analyzed (*6*). We found 33 *E. coli* and 35 *K. pneumoniae* ESBL-producing isolates in 55 samples (12 samples had >1 unique isolate), a total of 37% of ESBL-harboring samples (Table).

**Table:** Characterization of *Escherichia coli* and *Klebsiella pneumoniae* ESBL-producing isolates, Barrow, Alaska, USA*

Isolates, no.†	*bla* genotype				MLST profile
	CTX-M	SHV	TEM		
*E. coli*					
12	14	–	1		ST38 (ST2253)‡
11	14	–	–		ST131 (ST10)§
5	–	–	19		ST2967¶
3	27	–	–		ST405
1	15	–	–		ST131
1	–	–	1		ST2967¶
*K. pneumoniae*					
4	15	12	1		ND
5	–	12	1		ND
2	–	12	–		ND
2	–	102	19		ND
8	–	102	–		ND
1	–	–	19		ND
4	15	1	1		ND
1	–	2	–		ND

*ESBL, extended-spectrum β-lactamase; MLST, multilocus sequence type; ST, sequence type; ND, no data. †*E. coli* comprised 33 isolates from 32 samples. *K. pneumoniae* comprised 35 isolates from 35 samples. and 12 samples were both *E. coli* and *K. pneumoniae* ESBL-harboring isolates but did not display horizontal transfer resulting from deviating resistance genotypes. ‡One of the isolates harbored a novel MLST allele, giving the novel ST2253 (deposited in the *E. coli* MLST database at the ERI, University College, Cork, Ireland, UK (http://mlst.ucc.ie/mlst/mlst/dbs/Ecoli/). §One of the isolates had ST10; the remaining 10 had ST131. ¶Isolates harbored a novel MLST allele, rendering the novel ST2967 (deposited in the MLST database). The single isolate with only *bla*_TEM-1_ may contain undetected ESBL genes because of the non-ESBL phenotype of TEM-1.

We performed multi-locus sequence typing (MLST) on ESBL-producing *E. coli* isolates (*4*). Isolates were of described sequence types (STs) (ST131 [12 isolates], ST38 [10], ST405 [3], and ST10 [1]), and of previously undescribed STs (designated ST2253 [1 isolate] and ST2967 [6 isolates]) (Table).

In our 2005 study in Barrow, general resistance was relatively low, and no ESBL was found; surprisingly, however, 2 VRE isolates of a human clonal lineage were found (*3*; M. Drobni et al., unpub. data). Since then, resistance dissemination, particularly that of ESBLs, has exploded globally (*1*). In 2010, we found a high level of general resistance; 48% of randomly selected *E. coli* isolates displayed resistance toward >1 antibacterial drugs. This level is similar to the level we found in gulls in France in 2008, an area with high current and historical clinical antibacterial drug use and where birds have close contact with human activities (*4*).

We screened samples for VRE and ESBL-producing bacteria. The prevalence of VRE decreased from 6% in 2005 to 4.7% in the current study (*3*), indicating a slow decline or stability in VRE. ESBL, on the other hand, was not found in the 2005 study (M. Drobni et al., unpub. data) but emerged in 37% of samples carrying *E. coli* and/or *K. pneumoniae* harboring ESBLs. In the study from France, only 9.4% of birds carried ESBLs (*4*), although a study of gulls in Portugal during 2007–2008 reported an ESBL carriage of 32% (*7*), more similar to results of our current study but in contrast also because they investigated gulls from a highly populated area.

*E. coli* isolates mainly carried *bla*_CTX-M-14_ or *bla*_TEM-19_, whereas *K. pneumoniae* isolates mainly carried *bla*_CTX-M-15_, *bla*_SHV-12_, or *bla*_SHV-102_. To our knowledge, ESBLs in *E. coli* and *K. pneumoniae* have not been reported from Alaska, but in two 10-year perspective reports from Canada (*8*,*9*), similar patterns and genotypes are reported in *E. coli* and *K. pneumoniae* in clinical isolates (mainly from samples of persons with urinary tract infections and urosepsis). Our MLST of *E. coli* indicated 4 known STs; ST10, ST38, ST131, and ST405, all very common in the material from Canada (*8*), and major STs responsible for CTX-M dissemination worldwide (*1*). Two novel STs were found; several isolates were designated to 1 of them. We conclude that the relatively limited variation in clonal variants (STs) and ESBL genotypes is a consequence of recent introduction from connecting areas, such as Canada, possibly directly by bird migration or human activities, of a few resistant clones, followed by a local clonal expansion. This conclusion is supported by our 2005 study showing no ESBLs and by studies showing where different clones might have been introduced continuously for long periods, such as our study in France (*4*), which display a much larger diversity.

The dissemination of ESBLs to Barrow is part of this global pattern, and it is safe to say that humans and wildlife share resistant *E. coli* flora. When areas such as remote parts of Alaska are affected, global coverage is imminent.

Technical Appendix Resistance profile of randomly selected *Escherichia coli* isolates.
