# Supplementary material for: Extended-Spectrum β-Lactamases in Escherichia coli and Klebsiella pneumoniae in Gulls, Alaska, USA
Source: Emerg Infect Dis. 2014 May;20(5):897–9. doi: 10.3201/eid2005.130325 (PMC4012786; doi:10.3201/eid2005.130325)
Supplement: Technical Appendix — Resistance profile of randomly selected Escherichia coli isolates. [file 13-0325-Techapp-s1.pdf]

Article DOI: http://dx.doi.org/10.3201/eid2005.130325.

Extended-Spectrum β-Lactamases in *Escherichia coli and Klebsiella pneumoniae* in Gulls, Alaska, USA

Technical Appendix

Technical Appendix Table. Resistance profile of randomly selected *Escherichia coli* isolates*

	Resistance frequency		Co-resistance frequency		
Antibacterial drug	No. samples†	% of total	No. antibacterial drugs‡	No. samples†	% Total
Ampicillin	62	45	0	71	52
Tetracycline	10	7.3	1	44	32
Cefadroxil	8	5.8	2	18	13
Trimethoprim/sulfamethoxazole	6	4.4	3	1	0.7
Streptomycin	5	3.6	4	1	0.7
Nalidixic acid	4	2.9	5	1	0.7
Chloramphenicol	1	0.7	6	1	0.7
Tigecycline	0	0	>1	66	48
Nitrofurantoin	0	0			
Mecillinam.	0	0			

*Resistance was determined by antibacterial disk diffusion in accordance with recommendations from The European Committee on Antimicrobial Susceptibility Testing (EUCAST) (www.eucast.org). For antibacterial drugs lacking defined breakpoints for *E. coli* (tetracycline and streptomycin), the normalized resistance interpretation method (*1*) used by EUCAST, was implemented to define a local breakpoint.
†Total number of randomly selected *E. coli* was 137, isolated from 150 viable samples.
‡Denotes number of simultaneous antibacterial resistance phenotypes in each isolate.

Reference

1. Kronvall G. Kahlmeter G, Myhre E, Galas MF. A new method for normalized interpretation of antimicrobial resistance from disk test results for comparative purposes. Clin Microbiol Infect. 2003;9:120–32. PubMed http://dx.doi.org/10.1046/j.1469-0691.2003.00546.x
